# Twentieth century carbon stock changes related to Piñon-Juniper expansion into a black sagebrush community

**DOI:** 10.1186/1750-0680-8-8

**Published:** 2013-09-05

**Authors:** Daniel P Fernandez, Jason C Neff, Cho-ying Huang, Gregory P Asner, Nichole N Barger

**Affiliations:** 1 Department of Geological Sciences and Department of Environmental Studies, University of Colorado, 2200 Colorado Avenue, Boulder, CO 80309, USA; 2 Department of Geography, National Taiwan University, Taipei 10617, Taiwan; 3 Department of Global Ecology, Carnegie Institution for Science, 260 Panama Street, Stanford, CA 94305, USA; 4 Department of Ecology and Evolutionary Biology, University of Colorado, Boulder, CO 80309, USA

## Abstract

**Background:**

Increases in the spatial extent and density of woody plants relative to herbaceous species have been observed across many ecosystems. These changes can have large effects on ecosystem carbon stocks and therefore are of interest for regional and national carbon inventories and for potential carbon sequestration or management activities. However, it is challenging to estimate the effect of woody plant encroachment on carbon because aboveground carbon stocks are very heterogeneous spatially and belowground carbon stocks exhibit complex and variable responses to changing plant cover. As a result, estimates of carbon stock changes with woody plant cover remain highly uncertain. In this study, we use a combination of plot- and remote sensing-based techniques to estimate the carbon impacts of piñon and juniper (PJ) encroachment in SE Utah across a variety of spatial scales with a specific focus on the role of spatial heterogeneity in carbon estimates.

**Results:**

At a plot scale (300 m^2^) areas piñon juniper (PJ) encroached areas had 0.26 kg C m^-2^ less understory vegetation carbon compared to un-encroached sites. This lower amount of carbon was offset by an average of 1.82 kg C m^-2^ higher carbon in PJ vegetation and 0.50 kg m^-2^ of C in PJ surface-litter carbon. Soil mineral carbon stocks were unaffected by woody plant cover and density. Aboveground carbon stocks were highly dependent on PJ vegetation density. At a 300 m^2^ plot-scale, plots with low and high density of PJ forest had 1.40 kg C m^-2^ and 3.69 kg m^-2^ more carbon than the un-encroached plot. To examine how these 300 m^2^ variations influence landscape scale C estimates, historical and contemporary aerial photos were analyzed to develop forest density maps in order to estimate above ground PJ associated C stock changes in a 25 ha area. This technique yielded an average estimate of 1.43 kg m^-2^ of C accumulation with PJ encroachment. Combining this estimate with analysis of tree growth increments from dendrochronologies, we estimate that these PJ stands are accumulating aboveground C at an annual rate of 0.02 kg C m^-2^ with no slowing of this rate in healthy PJ. This result is in contrast to what has been observed in large areas of drought related PJ mortality, where C accumulation has ceased.

**Conclusions:**

These results illustrate that the encroachment of PJ forests in SE Utah over the last century has resulted in a large (and ongoing) accumulation of carbon in PJ trees and surface litter. However, the magnitude of the increase depends to on the density of vegetation across the landscape and the health of forest stands. Both management activities that remove forest carbon and forest mortality due to drought or wildfire have the potential to quickly reverse the multi-decadal accumulation of carbon in these stands.

## Introduction

Over the last century, increases in the spatial extent (‘expansion’) and density (‘thickening’) of woody plants relative to herbaceous species has been observed across a range of climatic, geologic, and topographic settings [1-3]. These physiognomic changes have impacted the structure and function of ecosystems in a number of ways. These changes include declines in herbaceous species diversity and productivity and alterations in the magnitude and spatial distribution of soil nutrients and water [3-11]. Changes in woody plant extent and density can have a large influence on ecosystem carbon storage. In general, woody proliferation leads to the accumulation of woody carbon biomass and estimates of the carbon uptake associated with woody encroachment suggest these processes could be responsible for up to 25% of the total US C sink [12-15]. Despite this potential for carbon storage, the implication of woody biomass changes on carbon stocks are highly uncertain and influenced by variety of conditions including the vegetation composition prior to encroachment, soil characteristics, disturbance regimes, land management, and climatic factors [16]. As a result there is a clear need to better understand the processes associated with carbon stock changes during woody encroachment and to improve the measurement of carbon stock changes in order to better predict how these regional vegetation changes affect national C inventories.

Estimates of changes in ecosystem C with woody encroachment require an understanding of how both above and belowground C responds to landscape changes associated with encroachment. However, environmental controls on above and belowground C may be highly variable and, at present, there are no simple relationships that can be used to predict how ecosystem C will change with encroachment [17-19]. Recent studies suggest that mean annual precipitation (MAP) across a range of growth forms (i.e. trees, arborescents, and shrubs) is positively correlated with carbon changes following encroachment. However, this relationship breaks down in arid and semi-arid ecosystems where woody encroachment can lead to both sequestration and a net release of C [15,17-19].

There are two major factors that complicate prediction of carbon stock changes following woody encroachment in arid and semi-arid ecosystems; first, soil carbon stock responses are highly variable across biomes and second, the high degree of ecosystem spatial heterogeneity within these ecosystems makes it challenging to accurately estimate large-scale carbon stock changes. Reviews of woody encroachment-associated SOC change indicate highly variable soil responses ranging from −40 and +120 percent across a range of US ecoregions [17,19,20]. Some of this variation is likely due to climatic and edaphic factors but some is also almost certainly associated with changing spatial patterns of woody and non-woody plants in ecosystems undergoing vegetation change.

Arid and semi-arid ecosystems tend to have heterogeneous and clumped vegetation distributions and the heterogeneity of above and belowground resources, including carbon, tend to increase when shrub and forest cover increases in grassland-dominated ecosystems [4-7]. Despite this spatial complexity, most studies of woody encroachment infer changes in C stocks from point measurements of above and below ground resources that do not incorporate information on the spatial distribution, density, or variation in aboveground biomass and how these changes relate to SOC [4-7,10,21]. Although these point-based measurements offer insight into the biogeochemical processes that influence carbon stability, they may not adequately capture the landscape-scale variation in woody plant density that may ultimately control ecosystem C stocks. For the studies that have examined the spatial distribution of woody plants, patterns of above-ground carbon appear related to the size and spatial distribution of tree and shrub canopies whereas belowground carbon can be influenced by the presence of canopy and plant stems [21-24]. This variability (and associated complexity in sampling design) presents a major challenge to ecosystem-scale estimates of carbon stock changes following woody encroachment.

In a number of regions across the western US, there have been widespread changes in the cover of piñon (*Pinus edulis, P. monophylla*) and juniper (*Juniperus osteosperma, J. monosperma*) (henceforth P-J). Over much of the 20th century, these trees have expanded into the semi-arid grasslands and shrublands of the western US [25-30]. More recently, a multi-year drought has lead to widespread (but patchy) mortality of these trees, setting the stage for a potential grassland return [31-33]. Here we examine the carbon stock changes associated with changes in PJ woodland cover in Southeastern Utah. We specifically focus on the question of how above and belowground carbon stocks change following PJ encroachment and subsequent drought-induced dieback. We then compare plot-level C stock measurements to historical and contemporary aerial photographs to examine how vegetation heterogeneity (density differences) affects estimates of ecosystem scale carbon stocks and determine whether object-based image segmentation techniques can be used in combination with historical photo archives to document regional changes in woody plant cover and associated aboveground C stocks following woody plant expansion. Evaluation of belowground C stock changes are only conducted on the 0–10 cm depth; previous work investigating SOC on the sandstone derived soils of study region in this research show SOC to be greatest in the top 10 cm of the soil profile and that SOC decreases substantially with depth [34,35]. Additionally these soils have a surface organic layer of little depth (1-3 cm).

## Methods

### Site description

The study site was located on the Colorado Plateau, specifically in the Hop Creek area within the Manti-La Sal National Forest on the southwestern flank of Shay Mountain (4196245, 622840; UTM NAD 83 zone 12 N). The site is located at 2000 m above sea level and characterized as an Upland Shallow Loam Black Sagebrush ecological site [36]. Precipitation in this study site averaged 393 mm yr^-1^ with a mean annual maximum temperature of 15°C and mean annual minimum of 2°C (http://www.wrcc.dri.edu/summary/climsmut.html) between the years 1971–2000. The site has been historically grazed by cattle and is currently grazed at an intensity of 145 AUM [37]. All analyses for this study were conducted on soils derived from the Kayenta Sandstone Formation [38]. Soils were classified as Barx fine sandy loam/fine-loamy, mixed, on mesic, Ustollic Haplargid [39] (see Table 1 for soil descriptions).

**Table 1:** Study area soil characteristics for each study plot

**Plot**	**Sample number**	**Mineral bulk density (g cm**^**-3**^**)**	**Duff bulk density (g cm**^**-3**^**)**	**Duff depth (cm)**	**Soil CO**_**3**_**(%)**	**Clay (%)**	**Silt (%)**	**Sand (%)**
HSC	32	1.55 (0.02)	0.13 (0.004)	0.38 (.03)	1.97 (0.69)	7.47 (0.01)	43.51 (O.09)	49.01 (0.11)
LD	34	1.38 (0.02)	0.25 (0.03)	1.53 (0.45)	1.17 (0.04)	8.23 (0.08)	37.35 (0.10)	54.41 (0.09)
HD	41	1.29 (0.02)	0.27 (0.04)	2.13 (0.35)	2.99 (0.84)	9.71 (0.09)	36.78 (0.11)	53.01 (0.14)
PJD	34	1.38 (0.02)	0.22 (0.02)	2.09 (0.46)	0.96 (0.07)	8.24 (0.07)	37.35 (0.11)	54.40 (0.17)

All data are calculated means with ± standard errors in brackets. Means for soil texture are based on an N of 6 per plot. Bulk density and soil carbonate values are based on the 0-10 cm soil depth.

**Figure 1:**
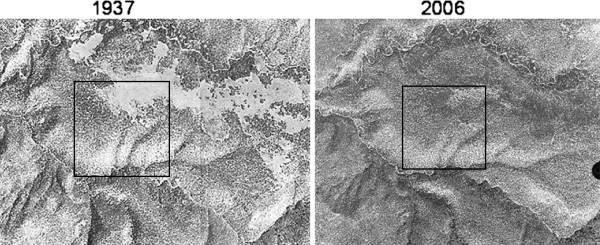
**Hop Creek study area aerial photos from 1937 and 2006.** The 1937 photo shows our study plots. The boxed areas in each photo show a 25 ha area where our study plots were located and was the area used for the image segmentation ortho-photo analysis.

The Hop Creek area was chosen because comparison of historical aerial photographs from 1937 (Figure 1) show significant *Pinus edulis* Engelm. (piñon) and *Juniperus osteosperma*(Torr.) (juniper) encroachment into a black sagebrush ecological site. The dominant vegetation in the unencroached area was comprised primarily of black sagebrush and the grasses *Achnatherum hymenoides, Bouteloua gracilis, and Sporobolus cryptandrus*. All field-based measurements described below were carried out in September 2006 in plots with similar slope position and aspect.

### Plot based C stocks

To quantify changes in C stocks associated with PJ expansion into a black sagebrush ecological site we established four 300 m^2^ plots (15 × 20 m) that differed in the degree of tree density and canopy cover. One plot was established in an area free of encroachment representing the historic sagebrush condition (HSC) prior to encroachment. Another plot was established in an area with low tree density (LD) and a third plot was established in high tree density (HD). Tree density and canopy cover differences between the LD and HD plots represent the degree to which PJ recruitment and establishment occurred over the past century within the Hop Creek area (Figure 1). As result, the names LD and HD are based on differences in tree density and canopy cover. Because we were also interested in the impact of the tree mortality on C storage, a fourth plot was established where there was significant piñon dieback (PJD).

Within each plot, we estimated cover of sagebrush, grasses, litter-duff, bareground, and tree basal canopy area (BCA). We also estimated aboveground (sagebrush, grass, and tree) and soil (organic and mineral layer) C stocks within each plot. Plant cover and above and belowground C stock changes as a result of PJ encroachment and PJ mortality were determined by comparing the LD, HD, and PJD values to that of the HSC plot.

To evaluate whether soil C stock spatial distribution changes reflect encroaching tree spatial distribution, we geo-referenced all trees and soil samples taken from the LD, HD and PJD plots and compared their distributions to that of the HSC plot. Additionally, trees from each plot were cored and dated in order to determine tree C accumulation rate within the study plots. Method specifics for each measurement are described below.

### Cover estimates

Fractional cover of sagebrush, grass, litter-duff, and bare-ground was estimated visually in 48, 1 m^2^ subplots within each 300 m^2^ plot. These subplots were evenly spaced throughout the 15 × 20 m 300 m^2^ plot whereby a 5 m^2^ subplot within the entire plot would have four 1 m^2^ subplots for cover estimation. These cover estimate subplots were located in the corners of each 5 m^2^ subplot of the 300 m^2^ plot. Tree cover estimates were determined for the LD, HD, and PJD plots by measuring the BCA of every tree in each plot. For large trees, BCA was determined by running a field tape around the tree canopy edge to estimate canopy circumference. Canopy circumference was then converted to a BCA. For small trees BCA was also determined by converting circumference to area. However, small tree circumference was determined by measuring canopy diameter along two perpendicular lines. All cover estimates are expressed on a 1 m^2^ basis.

### Carbon measurements

Carbon stocks of the understory grass and shrub functional groups was determined by converting cover values of each functional group to a standing stock of C. This was accomplished in two steps. First, functional group specific linear regression equations (Table 2) were developed to convert the fractional cover of grass and sagebrush into biomass. Linear regression equations used to calculate grass (r^2^ 0.96 p < 0.01) and sagebrush (r^2^ 0.90 p < 0.01) biomass were developed by harvesting aboveground plant material from eight 1 m^2^ plots with a 5 to 85 percent sagebrush or grass cover range. Harvested sagebrush wet weight was recorded in the field for each plot. Sub-samples of field weight sagebrush and all grass aboveground biomass were transported back to the lab and oven dried at 60°C for 48 hours in order to determine a dry weight. Dry weight was used to develop the linear regression equations for estimating sagebrush and grasses biomass as a function of percent cover. One square meter plots within 5 m^2^ subplots were averaged and scaled to represent the cover within the 5 m^2^ plot that they were located in. These 5 m^2^ averages were used to calculate sagebrush and grass biomass within each 300 m^2^ plot. Sagebrush and grass biomass was then converted into kg C by multiplying biomass by the mean percent C content of each plant (Table 2). Average sagebrush and grass percent C was determined by homogenizing and grinding five samples of each plant type with a shatterbox and analyzing it with an EA 1110 CNS combustion analyzer (Thermo Electron Corporation, Waltham, MA). Sagebrush and grass kg C estimates for each plot were presented on a 1 m^2^ basis.

**Table 2:** **Regression equations and *r***^**2**^**values used to calculate grass, black sagebrush, live PJ and dead PJ biomass and the percent carbon (C) within each plant type for determining kilograms of C**

**Plant type**	**Model**	***r***^**2**^	**% C**
Grass	Biomass = 0.566 * % cover m^-2^	0.96	40.84 (0.41)
Black sagebrush	Biomass = 12.132 * % cover m^-2^	0.90	45.55 (0.51)
Live Piñon	Biomass = 11.41 * (RCD * 0.1)^^2.6664^)	NA	46.14 (0.43)
Juniper	Biomass = 8.256 * (RCD * 0.1)^^2.8058^)	NA	45.64 (0.54)
Dead Piñon	Biomass = (Live Biomass - (1.853 *(RCD * 0.1)^^2.0268^))	NA	46.14 (0.43)

The methods for determining tree C were based on previously published allometric equations [40,41]. To calculate tree C, we measured root collar diameter (RCD) on all trees within the LD, HD, and PJD plots. Root collar diameter is the stem diameter at ground level and is used with allometric equations to calculate tree biomass [40,41]. For all woodland plots, RCD calculated tree biomass was converted to kg C by multiplying PJ biomass with plant-specific percent-C content values (Table 2). Piñon and juniper C content was determined from harvested, oven dried and ground PJ saplings as done for black sagebrush and grass. *P. edulis* and *J. osteosperma* specific allometric models developed by Darling in 1967 [40] were used to calculate piñon and juniper biomass. For dead trees devoid of needles, the green biomass portion of the allometric equation was subtracted from dead trees. Green biomass for dead trees was estimated using piñon- and juniper-specific green biomass allometrics developed by Darling in 1967 [40]. The Darling 1967 models [40] were used because we did not develop RCD tree biomass allometry equations specific to our study area. The Darling allometry equations were also used because they are for similar tree species and were developed in an area similar to Hop Creek. We also felt that the Darling equations used were more accurate estimators of biomass than those developed for estimating biomass at the national scale i.e. [42].

For soil C estimates, soil samples were collected every 2.5 m and geo-referenced. However, in plots with PJ encroachment, sampling points were collected 2–2.5 m in order to get a better spatial representation of overstory C inputs and better assess soil C stocks. The differences in sample spacing are addressed in the geostatistical analyses described later. At each sampling point, the duff/litter layer was collected when present with a large flat spatula and a circular piece of polyvinyl chloride (PVC) with a diameter of 10 cm. The depth of each duff/litter layer sample was recorded in order to calculate the bulk density of samples collected from this layer. Mineral soil samples were taken with a volumetric soil sampler (Soil Moisture Equipment Soil Core Sampler Model 0200). Ten cm long mineral soil samples were collected from the point where the duff/litter layer was collected. All soil samples were transported back to the lab and oven dried at 60°C for 48 hours. After drying, mineral soil samples were passed through a 2 mm sieve. This material was then passed through a soil splitter to obtain 2, 2 g sub-samples, which were then ground to a fine powder using a mortar and pestle. Sub-samples of soil were then acidified with 15% HCL in order to remove carbonates. Duff samples also had carbonates removed by this acidification process. Percent C content for mineral soil and duff samples were analyzed with an EA 1110 CNS combustion analyzer. All percent C values were converted to kg C m^-2^ using bulk density values for statistical and computational analyses. However prior to converting to kg C m^-2^ all percent C values were carbonate corrected with soil sample specific carbonate values. Soil specific carbonate values were determined by using a modified pressure calcimeter method [43].

Mineral and duff/litter SOC were analyzed separately in order to better understand soil C differences within different C pools. Because we wanted to spatially represent soil variability in our study, we collected a different number of soil samples within each plot and capture vegetation heterogeneity, the sample size varied somewhat across the plots (Table 1). Due to the differences in sample size, we used the non-parametric Kruskal-Wallis one-way analysis of variance (ANOVA) by ranks to determine whether SOC differed between plots. The geometric mean from each Kruskal Wallis test was determined to be statistically different by applying the multiple mean rank comparison. This analysis should be used primarily as a statistical reference for comparison of within plot variance vs. the site means. Our design does not allow for a true ANOVA analysis (e.g. there is only one true plot) and this analysis is used here as if our subplot measurements were independent samples (noting that many experimental designs use this approach without the caveats) and should be interpreted as such. All means from the Krustal Walis ANOVAs are followed by a bracketed standard error value.

### Spatial distribution of soil C and trees

The spatial distribution of soil C was evaluated with Moran’s Index for spatial autocorrelation (MI). Moran’s *I* measures the spatial autocorrelation of geo-referenced values and evaluates whether their distribution is clustered, dispersed, or random. The spatial distribution of trees was evaluated with the average nearest neighbor (ANN) measure. Unlike MI, which measures the spatial distribution of values, ANN measures the spatial distribution of objects. For MI and ANN a value near +1.0 indicates clustering, a value near −1.0 indicates dispersion, and a value near zero indicates a random distribution. Each calculated MI and ANN output has a calculated z-score. The statistical significance of a clustered or dispersed distribution is determined by comparing z-scores to a range of confidence intervals. For example, in order for a clustered or dispersed distribution to be statistically significant at a *p* < 0.05 or 0.01, the z-score has to be respectively between −1.96 and 1.96 or −2.58 and 2.58.

### Tree age

We evaluated tree age structure of piñons within each of the plots to examine tree recruitment rates over the last century. Tree recruitment data was used to examine annual aboveground C accumulation with the occurrence of tree encroachment. An increment core was collected from all piñons in each plot to determine tree age. We did not collect increment cores from *J. osteosperma* due to known difficulties in dating this species. Total tree C values were then divided by tree age in order to calculate a kg C yr^-1^ accumulation rate for each tree. In this analysis we use tree data from LD, HD, and PJD plots; however, PJD trees were treated as living trees with green biomass C at the time of death. Tree cores were collected from all piñons > 5 cm in diameter. Tree cores were collected approximately 10–15 cm above the ground. Tree cores were analyzed at the Institute for Arctic and Alpine Research’s Dendrochronology Lab, University of Colorado, Boulder. Increment cores were mounted [44] and progressively sanded with FEPA (Federation of European Producers of Abrasives) 120-, 220-, 320- and 400-grit (162, 68, 44.7-47.7 and 33.5-36.5 μm respectively [45], sandpaper using a Bosch belt sander (Robert Bosch Corp., Farmington Hills, MI.) Tree cores were also hand-surfaced using 400 and 1200-grit sandpaper (33.5-36.5 μm). Tree cores were then visually cross-dated using a previously developed piñon chronology for NMM (C. Woodhouse, unpublished data). For samples difficult to visually crossdate, undated inner sections were run against the previously developed chronology using COFECHA [46]. COFECHA is a software program used to check the quality of the cross-dating. All matching dates found using the COFECHA program were then visually verified. We recorded the first year of secondary growth to calculate tree age. Although we made 3 to 5 attempts in the field to obtain cores that included pith, we were not always successful. We estimated dates for cores without pith by overlaying sets of concentric circles on the inner rings of the core [47]. To correct for the time to coring height, we collected piñon seedlings from a range of sites and soil types across the region. Average time to a coring height of 15 cm is approximately 11 years. Once an inner ring date was obtained from the cores, 11 years was subtracted from this date to correct for the time to coring height.

Coupling tree age with aboveground C data, we modeled annual changes in aboveground C in the LD, HD, and PJD plots from the late 1800s through 2005. To estimate an annual increase in tree C, total tree C was divided by tree age in order to estimate an average annual C increase for each year of growth. Although factors such as climate and tree age clearly influence annual growth, based on observation of annual growth rings there was no simple model (eg. negative exponential) that could be applied to annual tree growth at our sites. Thus we chose to increase individual tree C by an average amount for each year. Since junipers were not dated, we used a linear regression equation of tree age as a function of root collar diameter from a previous study in the region (Barger, unpublished results) to estimate juniper age within each plot (Juniper Age = 2.82(RCD) + 19.8, *r*^2^ = 0.62). Initial conditions for pre-encroachment aboveground C was set at the level of aboveground C estimated from the HSC plots. The starting year for aboveground tree C accumulation for each of the plots was determined by the oldest tree in each plot (1891, 1906, and 1901 for LD, HD, and PJD respectively). In addition to accumulation of aboveground C in the tree layer, we estimated declines in belowground C in the understory layer over time. Tree density in the four plots was a strong predictor of understory biomass (Understory C = −0.516 * Tree Density + 137.9, *r*^2^ = 0.84). Following this, we estimated changes in understory C over time as a function of annual historical tree density based on tree recruitment into the plots. Historical tree density was used to estimate changes in aboveground understory C.

In this analysis we use tree data from LD, HD, and PJD plots. PJD trees were treated as living trees in the aboveground biomass accumulation until the year of death. After the year of death we assumed that all living aboveground biomass in the PJD plots was converted to dead biomass.

### Photo analysis of tree C estimate

The purpose of the aerial photo-analysis was two fold; 1) assess the feasibility of utilizing aerial photography for the estimating aboveground C over large areas by comparing plot and aerial based estimates and 2) to determine tree C accumulation as a result of encroachment between 1936 and 2006. To accomplish this task we used an object-based image segmentation technique capable of detecting individual (or clumped) tree canopies (henceforth image segmentation) (ENVI Feature Extraction module, ITT Visual Information Solutions, Boulder, CO) to delineate woody canopies. These canopy areas were then imported into a GIS system (ArcGIS v. 9.3, ESRI, Redlands, CA) to calculate canopy area (CA) [48]. We applied previously published remotely sensed aboveground C calculations to calculate tree C from canopy area [49]. The spatial extent of this analysis was a 25 ha area encompassing our study plots. A single allometric tree biomass equation that uses CA and developed by Huang et al. [49] was used to calculate tree biomass”

$$\log_{10}\text{biomass}\left(\mathrm{kg}\right)=-4.66+1.{32\log}_{10}\mathrm{CA}\left({\mathrm{cm}}^{2}\right)$$

The CA based allometric equation by Huang et al. [49] grouped the RCD models developed by Darling in 1964 [40] for *P. edulis* and *J. osteosperma* into one because these species have no structural difference when CA is used to estimate aboveground biomass in this study region. Tree C was calculated by multiplying biomass by 0.46; the average percent C of piñon and juniper plant material.

To estimate C accumulation in the delineated 25 ha area, new canopies were determined by overlaying the 1937 and 2006 DOQQ aerial photographs. For this analysis the quality of 1937 aerial photograph (Figure 1) was suitable for visual inspection but insufficient for quantitative image segmentation analysis. As a result, visible, open, woody plant free areas were delineated by on-screen digitizing in ArcGIS. The woody plants (and aboveground C) derived from 2006 DOQQ located in these 1937 woody plant free areas were summarized to represent the amount and rate of C accumulated due to PJ encroachment within the 25 ha study area.

The plot-based estimates of aboveground C stocks were then compared to the image segmentation estimates of aboveground C stocks. Comparisons were first conducted by extracting image-segmented canopies within the LD and HD plots in order to detect tree C differences between our plot-based measurements and image segmentation derived values. We also scaled plot-based carbon stocks to the 25 ha study area and then compared these estimates to the carbon stocks determined through image segmentation.

## Results

Piñon pine was the dominant tree species in the LD and HD plots, comprising 88-96% of the tree population (Table 3). Piñon age across the study plots supports observations from aerial photos that recruitment into the open black sagebrush sites occurred in the early 1900s. Piñon age data within all plots indicates that trees were present as early as 1894 and that a pulse in recruitment occurred in the 1940s and 50s with 43–66% of the trees across all plots dating to these decades (data not shown). Average piñon age (excluding saplings) was similar across the PJ encroached plots and ranged from 47 – 53 years. Tree density in the HD plot was 47 percent higher relative to the LD plot (trees ha^-1^, HD = 1567; LD = 1067). Tree density in the PJD plot was similar to the LD plot with 1000 trees ha^-1^ (live + dead) but declined to 100 live trees ha^-1^ after the regional mortality event in the early 2000s (Table 3). Percent tree mortality within PJD was 90% and based on outer ring dates all tree mortality occurred in 2003, with the exception of one tree that died in 2004. The HSC plot contained four juniper saplings and no piñon trees.

**Table 3:** **Total number of trees per plot broken down by species, mean tree age, mean basal canopy area (BCA), tree trunk basal diameter (TCD), and percent grass, sage, duff/litter, and bareground cover per m**^**2**^

**Plot**	**Live tree density**	**Mean tree**	**Mean tree BCA (m**^**2**^**)**	**Mean TCD (cm)**	**Mean grass cover (%)**	**Mean sagebrush cover (%)**	**Mean duff/litter cover (%)**	**Mean bareground cover (%)**
	**(Stems ha**^**-1**^**) above**	**Age (yrs)**						
	**Number of trees below**							
HSC	133 4 (J saplings)	NA	NA	NA	11.85 (0.43)	37.03 (0.95)	NA	41.38
LD	1033 31 (30 P, 1 J)	51 (3)	4.2 (0.7)	10.64 (1.04)	11.97 (2.43)	25.62 (2.57)	27.60 (4.06)	31.98 (4.04)
HD	1400 42 (37 P, 5 J)	53 (2)	5.1(0.8)	13.70 (1.32)	2.71 (1.06)	14.16 (1.88)	53.84 (5.47)	27.08 (4.42)
PJD	100 30 (15 P, 3 J)	47 (1)	4.9 (2.5)	8.54 (1.55)	20.41 (2.32)	25.83 (2.25)	25.21(3.36)	24.58 (3.22)

For the HSC plot duff/litter and bare ground estimates are combined and presented in the bareground column. Percent cover estimates do not add up to 100 because forbs and shrubs other than black sagebrush are not included. Values in brackets are ± 1 standard error of the mean. *P* = *Pinus edulis*, *J* = *Juniperus osteosperma*.

**Figure 2:**
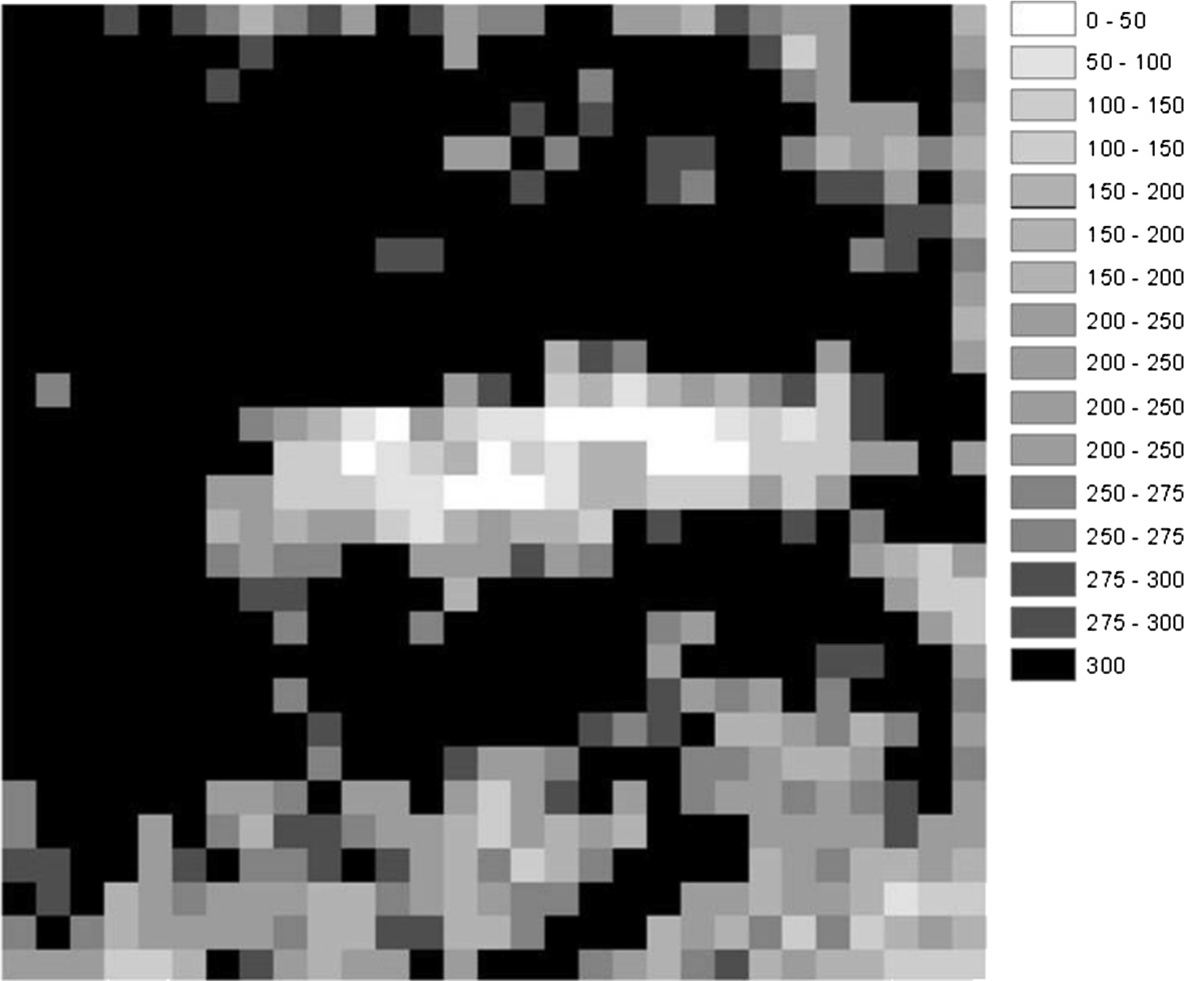
**2006 1-m resolution DOQQ of the of the Hop Creek area segmented into 300 m**^**2 **^**cells showing canopy area variance.** The color ramp represents m^2^ canopy cover for grid cells lying within the 25 ha image segmented area.

Evaluation of a 25 ha area in the 1937 orthophoto and 2006 DOQQ indicates that ~ 32% of the landscape was treeless (similar to the HSC). Additionally evaluation of the 2006 DOQQ shows that ~22% of the encroached area had less canopy cover than our HD plot when the 2006 DOQQ is segregated into 300 m^2^ sections (Figure 2). We did not attempt to map areas of dead PJ woodland in this analysis.

### Vegetation cover and C changes

Broad changes in understory cover and composition were evident with woody encroachment in these plots (Table 3). Live tree density was inversely related to understory (sagebrush and grass) cover. Sagebrush C stocks in the encroached sites were lower and corresponded to a decline of 0.1 kg C m^-2^ in the LD and PJD subplots and a decline of 0.24 kg C m^-2^ in the HD subplots with the HSC site as reference point (Table 4). Grass cover was 11.8 ± 0.4% within the HSC subplots (Table 3) accounting for a C stock of 0.006 ± 0.002 kg C m^-2^. Grass cover in the LD was similar to that of HSC, lower in the HD, and greater the PJD subplots (Table 4).

**Table 4:** **Total and individual kg m**^**-2 **^**C stocks for each plot**

**Carbon Stock**	**HSC (kg m**^**-2**^**)**	**LD (kg m**^**-2**^**)**	**HD (kg m**^**-2**^**)**	**PJD (kg m**^**-2**^**)**
Piñon and Juniper	0.0003 (NA)	1.13 (NA)	3.24 (NA)	1.10 (NA)
Black sagebrush	0.41 (.01)	0.31 (0.03)	0.17 (0.02)	0.31 (0.02)
Grass	0.006 (.002)	0.006 (0.001)	0.001 (0.0006)	0.011 (0.001)
Duff/Litter	0.05 (0.01) c	0.42 (0.11) b	0.71 (0.13) a	0.53 (0.14) a
Mineral Soil	1.52 (0.06) ab	1.68 (0.07) a	1.37 (0.05) b	1.44 (0.06) b
Total C	1.99	3.55	5.49	3.39

Numbers in brackets are ± standard error values. For duff/litter and mineral soil values with the same letter are not significantly different. Piñon and Juniper C stocks are calculated with the allometric equations developed by Darling [40].

Total aboveground carbon was 140-712% higher n PJ encroached subplots relative to HSC subplots. Compared to the 0.42 kg m^-2^ of C in the HSC plot, the LD, HD, and PJD plots contained approximately 1.02, 2.99, and 0.99 kg more aboveground C per m^2^ respectively. These changes occurred despite declines in grass and sagebrush C.

### Litter and soil C stocks

Total soil C stocks were 32-34% higher in encroached subplots, which was driven by differences in the amount of C in the duff/litter layer (Table 4). The difference in duff/litter C is attributed to PJ encroachment because duff/litter in encroached subplots is comprised primarily of PJ needles and small PJ twigs. There were significant differences in duff/litter C between subplots (Kruskal Wallis: H = 15.56, p = 0.001). A multiple comparison of mean ranks for all groups test showed duff/litter C on a kg m^-2^ to be lower in the HSC subplots than the LD subplots and that duff/litter C is highest in the HD and PJD subplots.

Carbon stocks in the surface 0–10 cm of the mineral soil layer followed a different pattern from the duff/litter soil layer. Differences in mineral SOC did exist between the subplots but not in a consistent pattern and the magnitude of the differences were relatively small (Table 4; Kruskal Wallis: H = 12.68, df = 3, n = 141, p = 0.005). Mineral SOC respectively averaged 1.52 ± 0.06, 1.68 ± 0.07, 1.37 ± 0.05, and 1.44 ± 0.06 kg m^-2^ in the HSC, LD, HD, and PJD subplots.

### Spatial C distribution patterns

Piñon and juniper encroachment influenced the spatial distribution of the duff/litter but not the distribution of mineral layer SOC. Mineral layer SOC was randomly distributed in all plots (Table 5) regardless of aboveground vegetation cover or condition. Duff/litter C distribution was determined to be random in the HSC plot but clustered in the LD, HD and PJD plots. The clustered duff/litter distribution occurred in association to the spatial distribution of trees.

**Table 5:** The spatial distribution of mineral and the duff/litter soil carbon (C) and encroached plot trees

**Plot**	**HSC**	**LD**	**HD**	**PJD**
MI mineral soil C	−0.15	−0.01	−0.13	−0.01
Z-score MI mineral soil C	−0.88	0.09	−0.86	0.38
P-value MI mineral soil C	NA	NA	NA	NA
Distribution mineral soil C	random	random	random	random
MI duff/litter C	−0.04	0.32	0.22	0.25
Z-score MI duff/litter C	−0.05	2.7	2.1	2.2
P-value MI duff/litter C	NA	0.01	0.05	0.05
Distribution duff/litter	random	clustered	clustered	clustered
ANND tree	NA	0.61	079	0.59
Z-score ANND tree	NA	3.7	1.9	2.9
P-value ANND tree	NA	0.01	0.05	0.01
Distribution tree	NA	clustered	clustered	clustered

The spatial distribution of soil C pools is based Moran’s *I* (MI) for spatial autocorrelation. The spatial distribution is based on the Average Nearest Neighbor Distance (ANND) value.

### Aboveground C changes over time

**Figure 3:**
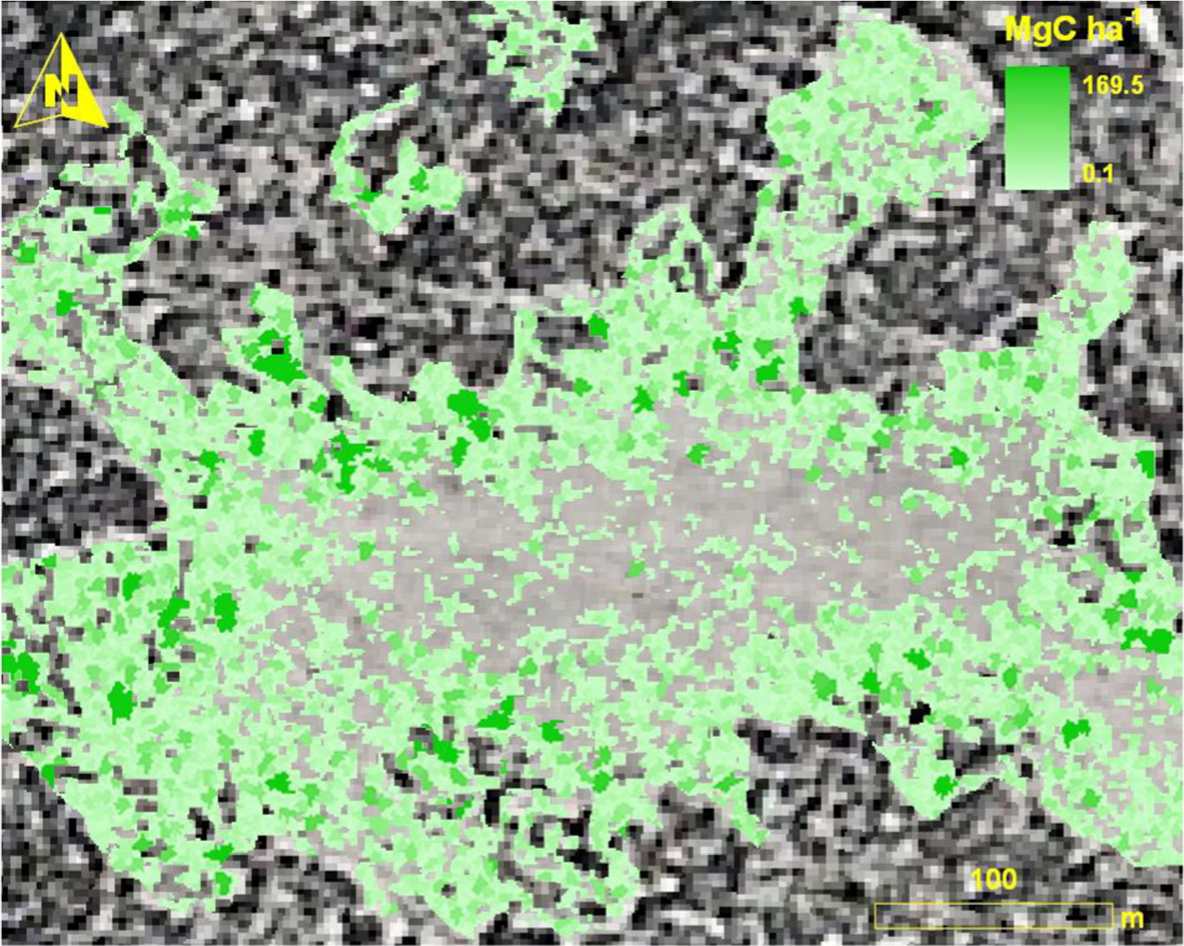
Modeled aboveground C change with tree encroachment.

Estimates of aboveground C stock changes in the encroached plots over the last century suggests that these upland PJ sites have a high capacity to rapidly accumulate aboveground C. Modeled aboveground C accumulation based on tree recruitment data suggests that aboveground C has exponentially increased over the past century (Figure 3). These exponential increases in aboveground C, however, did vary between the LD and PJD subplots relative to the HD subplots; a pattern likely driven by differences in tree density.

### Image segmentation photo-analysis tree carbon

Comparison of the 1937 photo and 2006 DOQQ shows that in 2006 PJ occupied ~68% of area that was open in 1937 (Figure 1). Image segmentation analysis delineated nearly the same number of canopies and canopy area as measured on the ground in the LD and HD plots (Table 6). This image segmented canopy area when converted to a C stock with canopy based allometry results in a stock estimate that is approximately 30% greater than what was directly measured in the LD plot and a 10% lower in the HD plot.

**Table 6:** **Object-based image segmentation and ground based measurement comparisons for the LD and HD 300 m**^**2 **^**plots**

**Measurement**	**LD**	**HD**
Image segmentation canopies	32	42
Ground measured canopies	31	42
Image segmentation canopy area plot^-1^ (m^2^)	137.00	235.00
Ground measured canopy area plot^-1^ (m^2^)	130.05	213.57
Image segmentation mean canopy area tree^-1^ (m^2^)	4.15 (0.55)	5.46 (0.59)
Ground measured mean canopy area (m^2^)	4.19 (0.69)	5.08 (0.83)
Image segmentation kg C plot^-1^	455.12	846.11
Ground based measured kg C plot^-1^ using Huang et al. [49] calculation	457.98	833.39
Ground based measured kg C plot^-1^ using dbh	327.26	937.49

Image segmentation tree C estimates per plot use the equation developed by Huang et al. [49]. Ground based tree C estimates are shown twice. Once using tree canopy area and the Huang et al. [49] equation and once using dbh and the appropriate tree species equation.

Comparison of ground and image-segmentation-based estimates of C/tree yielded very similar estimates. Tree C within the LD and HD plots measured 17.2 kg with ground based measurements and 17.7 kg with image segmentation. The differences between the methods are evident when scaling the directly measured values from the 300 m^2^ field plots to the larger 25 ha study area. A direct scaling of the LD and HD plots to 25 ha yielded estimates of 267 and 781 Mg C respectively. In contrast image segmentation analysis, which measures the entire 25 ha area yielded an estimate of 358.93 Mg C. Differences in the plot-level and remote sensing measurements occur because plot measures cannot account for the tree density variation in the larger woodland area; whereas the image segmented C estimate does incorporate canopy density woodland variation (Figure 2).

**Figure 4:**
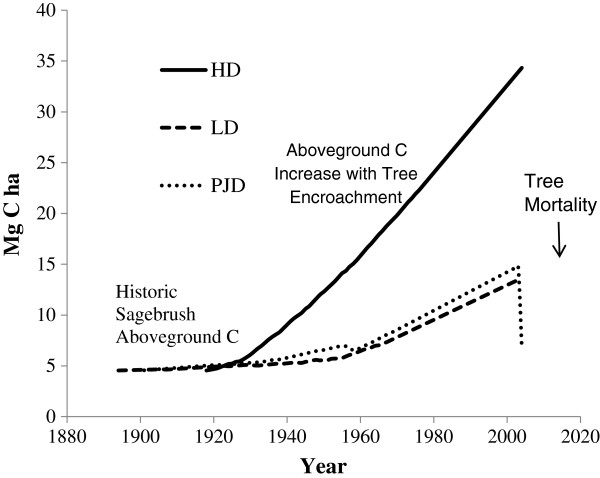
Woody C increase area and magnitude from 1937 to 2006 in the Hop Creek region.

Combining the remote sensing estimate C stocks with the tree ring analysis, we can estimate the rate of C increase in this area. We estimate that 869 trees ha^-1^ expanded into treeless areas between 1937 and 2006 (Figure 4) resulting in a woody C increase of 15.36 Mg C ha^-1^; and annual C accumulation increase of 0.22 Mg ha^-1^.

## Discussion

The expansion of piñon-juniper populations into ecosystems in SE Utah, USA, causes a predictable decline in grass and shrub cover in proportion to tree canopy cover. These changes in vegetation cover lead to the loss of historical vegetation C; however, the loss of historical vegetation C is offset by large increases in carbon associated with aboveground PJ growth and the accumulation of PJ surface-litter C. More notably, the changes in above ground carbon are highly variable across space indicating that the density of PJ woodlands in this region is the most important factor controlling the accumulation of C with woody encroachment.

### Woody encroachment and ecosystem C stocks

Trees represent the major carbon stock in these SE Utah woodlands and the density and recent establishment of PJ woodland varied across our study area (Figures 2 and 3). At the level of our experimental plots and in the larger analysis of aerial photographs, the spatial variation between relatively low and high density PJ woodland is responsible for the majority of the variation in C stock estimates within the Hop Creek study area. The net change in C between the historical condition and present states in the low-density and high-density plots respectively differs nearly threefold. This result highlights the issues of field-based methods in sampling carbon changes across this type of landscape. Had we simply sampled the LD and HD sites without attention to variation in density (e.g. in a long point-transect study), the mean value for these two sites would have been approximately 46% higher than our estimate for C based on analysis of orthophotos, which more accurately capture variation in tree density across this site. According to a one-correlation *t*-test power analysis, 258 300 m^2^ plots delineated within Figure 3 would need to be sampled in order to sample 90% of the variation within the 25 ha area.

The second major change in carbon stocks with PJ expansion is in the litter/duff layer. The spatial distribution of litter/duff C follows the distribution of trees and is proportional to tree density (Tables 3 and 5). The accumulation of relatively recently fixed and non-mineral or aggregate stabilized C is similar to findings from a site in Southern Utah where the bulk of soil C accumulation following PJ establishment occurred in what is the equivalent to the duff/litter layer studied here [21]. Further, the estimated turnover time of this surface C pool in southern Utah was on the order of 10–20 years [21], a relatively rapid turnover time. The potential for rapid turnover of duff/litter combined with the vulnerability of these forests to drought-related mortality (as shown in the recent death of PJ stands in this study area) highlights that the carbon gains associated with the expansion of these forests depend on the continued health and growth of these forests. Management activities that reduce forest biomass or disturbances such as drought induced tree mortality or fire can lead to carbon losses that quickly reverse carbon accumulation in these forests.

The carbon stock with the greatest inherent potential for long-term carbon stabilization is the mineral soil layer, where carbon could, in principle, be protected for decades to centuries. However, in this study, there were no appreciable differences in soil carbon stocks in areas with or without PJ forest. In these areas of SE Utah, the amount of carbon in the mineral soil layer is small. Additionally mineral layer soil C in soils similar to that of Hop Creek area that are mineral-stabilized only accounts for a small fraction of the soil C [21]. However, changes in soil carbon stocks with woody encroachment in other arid and semi-arid ecosystems differ from what was found in the Hop Creek area and suggests that soil C stock changes seem to be controlled by a number of confounding factors such as land-use history, past disturbance, and soil physical characteristics such as bulk density, texture, and mineralogy [17,19,34,35].

### The temporal dynamics of carbon accumulation

In the Hop Creek study area, the woodlands are still relatively young and appear to still be in a period of net C accumulation. Tree C accumulation rates for the plots examined here are rapid and increasing. For example, in the HD and LD woodland plots examined here, tree C accumulation averaged 0.37 kg tree^-1^ year^-1^. The analysis based on image segmentation predicted an average tree C accumulation rate of 0.22 kg tree^-1^ year^-1^; however this estimate is based on the 69-year time-span derived from the 1937 and 2006 aerial photos. Using the average tree age from the plot-based data for the estimation period the rate of C accumulation for new trees using image segmentation is 0.30 kg yr^-1^; relatively similar to the plot-based estimate. At this rate of increase, these forests are accumulating C at an annual rate of ~0.22 Mg ha^-1^. It is notable that this study indicates that carbon accumulation in these forests is continuing with little sign of decline over time. This result differs from other studies that document a slowing of the rate of C accumulation as trees age and forests thicken [31,50,51].

The continuing accumulation of carbon in trees is striking when compared against evidence for widespread PJ mortality across the southwestern U.S. and in these experimental sites [32,33]. During the multi-year drought of 2000–2005, large areas of PJ woodlands died [32] including the site identified here as PJD. Just as woodland encroachment shifts the distribution of C within ecosystem compartments, woodland dieback also results in a redistribution of material. In this study, this shift is seen in the comparison of the LD and PJD plots, which have similar duff/litter area cover but the PJD plot duff/litter layer is significantly deeper and richer in organic C (Table 4). The timing of dieback (~2005) and the apparent difference in duff/litter C stocks of 0.11 kg C m^-2^ suggests that approximately 10% of tree C was transferred from live biomass to the duff/litter soil compartment within a year of tree mortality. This observation, combined with evidence for rapid (decadal) turnover rates of surface, un-stabilized C stocks in nearby PJ ecosystems [21] suggests that a sizeable fraction of the 20th century sequestration of C due to PJ encroachment may be returned to the atmosphere due to recent tree-mortality in the next few decades. The combination of continuing carbon accumulation in growing PJ forests alongside patchy drought-related mortality further highlights the need to consider large-scale forest population dynamics in regional carbon stock estimates.

## Conclusions

The importance of tree density and heterogeneous PJ drought mortality on C stocks further highlights the need for high-resolution, large spatial scale analyses of C mapping. However, the limitation of these techniques is that they cannot be used to assess understory plant cover and soil C stocks. Changes in surface duff carbon and soil carbon in arid land ecosystems tend to be highly variable and most easily quantified by plot scale measurements. Additionally, the loss of pre-encroachment vegetation cover (and carbon stocks) is difficult to establish from aerial photos or other remote sensing analyses alone. In this site, if we relied solely on image segmentation analysis, we would estimate that PJ encroachment since 1937 resulted in a woody C increase of 15.36 Mg ha^-1^. However, our plot-based studies indicate that shrub C loss during woodland expansion accounts for approximately 2 Mg ha^-1^ of carbon loss thus lowering the carbon sequestration estimate by approximately 13%. None-the-less, this study illustrates the importance of cross-scale high-resolution analyses of woodland biomass distribution and the sensitivity of ecosystem C stock estimates to variation in tree cover density. The distribution and density of woodlands is a critical determinant of landscape-scale C stocks and apparently more important, than variation due to different tree allometry equations. The counterpoint to continued C sequestration in PJ ecosystems is the potential rapid and large loss of C due to large-scale mortality events. Our calculations suggest that as much as 25% of the C stabilized in the past century could be returned to the atmosphere in the next two decades in this regions woodland stands heavily impacted by drought-related mortality. Those losses could be larger if additional disturbances (e.g. wildfire) occur.

## Abbreviations

ANN: Average nearest neighbor; BCA: Basal canopy area; C: Carbon; COFECHA: Computer-assisted tree-ring chronology composition system; ha: Hectare; MI: Moran’s index; PJ: Piñon juniper; SOC: Soil organic carbon; RCD: Root collar diameter; N: Sample number.

## Competing interests

The authors declare that they have no competing interest.

## Authors’ contributions

All authors were involved in developing the field work protocols and conducted field sampling. All authors read and approved the final manuscript.
